# Device-Based Security to Improve User Privacy in the Internet of Things †

**DOI:** 10.3390/s18082664

**Published:** 2018-08-14

**Authors:** Luis Alberto Belem Pacheco, Eduardo Adilio Pelinson Alchieri, Priscila América Solís Mendez Barreto

**Affiliations:** Department of Computer Science, University of Brasília, Brasília 70910-900, Brazil; luisbelem@gmail.com (L.A.B.P.); pris@unb.br (P.A.S.M.B.)

**Keywords:** Internet of Things, cloud computing, fault tolerance, security and privacy

## Abstract

The use of Internet of Things (IoT) is rapidly growing and a huge amount of data is being generated by IoT devices. Cloud computing is a natural candidate to handle this data since it has enough power and capacity to process, store and control data access. Moreover, this approach brings several benefits to the IoT, such as the aggregation of all IoT data in a common place and the use of cloud services to consume this data and provide useful applications. However, enforcing user privacy when sending sensitive information to the cloud is a challenge. This work presents and evaluates an architecture to provide privacy in the integration of IoT and cloud computing. The proposed architecture, called **PROTeCt**—**P**rivacy a**R**quitecture for integrati**O**n of internet of **T**hings and **C**loud computing, improves user privacy by implementing privacy enforcement at the IoT devices instead of at the gateway, as is usually done. Consequently, the proposed approach improves both system security and fault tolerance, since it removes the single point of failure (gateway). The proposed architecture is evaluated through an analytical analysis and simulations with severely constrained devices, where delay and energy consumption are evaluated and compared to other architectures. The obtained results show the practical feasibility of the proposed solutions and demonstrate that the overheads introduced in the IoT devices are worthwhile considering the increased level of privacy and security.

## 1. Introduction

The adoption of Internet of Things (IoT) in diverse areas is significantly growing, and several platforms and services are being offered as part of this technology [1]. The IoT concept envisages the integration of varied types of objects (or “things”) within the Internet, providing useful information and enabling automation. The IoT enables an enormous range of applications, in many different areas, such as personal use (e.g., smart watches and health monitoring), governance (e.g., smart cities) and industrial (e.g., automation) [2].

The devices that comprise the IoT are very heterogeneous, ranging from devices with very constrained processing power and a limited energy source (battery) to fully fledged devices. Wireless Sensor Networks (WSN) are considered, among other technologies, to be an important enabler to IoT networks. WSN are composed of several small devices that have a processing unit, a sensor that enables interaction with the physical world and an antenna for wireless communication [3]. As the IoT adoption increases, the number of WSNs also grows as does the number of devices connected to the Internet and the amount of data generated by those devices. In order to store, process and deliver this amount of data in a suitable way, cloud computing has been successfully used [4,5,6,7,8,9]. The integration of IoT and the cloud provides the necessary scalability, since devices are only responsible for generating and sending data to the cloud. This approach also enables the use of generated information without constraints, such as device availability or processing power, i.e., even though the information is generated by a small constrained device, it can be consumed by a lot of services and users.

Although the integration of IoT and the cloud brings several benefits, it also creates some new challenges. Among those, a very important challenge is related to the storage of private information in a third party infrastructure (the cloud provider). In such a scenario, it is imperative that the architecture for this integration provides a way to ensure the user that privacy requirements are being met by a third party. This study improves the state of art regarding privacy in the IoT and cloud computing integration by presenting and analyzing several architectures that tackle this issue. Since the IoT is heterogeneous by definition, the analysis is conducted with severely constrained devices that comprise a WSN. Consequently, any device with more processing power is able to employ the studied architectures.

Several works have studied the integration of IoT and cloud computing, but most of them did not mention privacy. Some notable exceptions are the architectures proposed in SensorCloud [6] and UPECSI (User-driven Privacy Enforcement for Cloud-based Services in the Internet of Things) [8]. SensorCloud proposed a cloud-based architecture where privacy requirements are enforced by gateways, called Trust Points. In SensorCloud, all sensitive data is encrypted prior to being stored in the cloud platform, and only authorized entities have the key. This approach prevents the cloud platform from accessing the stored data—only services that will consume and present information have access to it. User-driven Privacy Enforcement for Cloud-based Services in the Internet of Things (UPECSI) [8] extends SensorCloud by implementing a privacy wide user-developer integrated solution. It includes a privacy development language (PDL), where the cloud service developer must describe how a user’s data can be used and enables the user to disable some service features according to its privacy requirements. In both these studies, the IoT network’s communication layer is abstracted, i.e., security and privacy are only ensured from the gateway and not inside the IoT network itself.

In an earlier version of this work [9], we briefly presented an architecture that extends UPECSI by transferring the privacy enforcement tasks from the gateway to the IoT devices. The resulting architecture is called **PROTeCt** (**P**rivacy a**R**quitecture for integrati**O**n of internet of **T**hings and **C**loud computing), and a preliminary evaluation showed its practical behavior [10]. PROTeCt adapts all communication protocols to consider the constrained nature of most IoT devices, extending the architecture protection spectrum, i.e., it decreases the attack surface since data leaves the IoT devices already protected. The main challenge is to implement this approach in an efficient and integrated way, i.e., avoiding overloading the IoT devices and, at same time, protecting the data in a way that cloud services can use it as needed/authorized. PROTeCt improves network security and reliability since (1) the gateway does not have access to plain data; thus, it can not impair the system security properties once compromised by a successful attack; and (2) a simple gateway crash does not impair the system’s availability, since the architecture does not contain this single point of failure. Although the gateway does not apply security and privacy schemes in the users’ data, one or more gateways could still perform other support tasks, such as protocol translation.

Regarding the architectures above described, this work presents at least four main contributions:

- It presents a detailed description of PROTeCt, a privacy aware architecture for the integration of IoT and cloud computing, extending the discussion presented in [9] and highlighting its main differences when compared to UPECSI [8].
- It discusses the advantages and disadvantages of these architectures regarding many aspects, like the assurance of security and privacy properties and overhead introduced at IoT devices by cryptographic functions.
- It presents an analytical analysis regarding the costs involved in implementing security and privacy mechanisms directly at the IoT devices.
- It presents a new experimental evaluation of these architectures, expanding previous results [10] and considering new scenarios. The evaluation is carried out through simulations conducted in the ns-3 network simulator, considering WSNs of different sizes (ranging from 4 to 160 devices) and using a solution without security as the baseline. The evaluation is based on the end-to-end delay and energy consumption (device life time). The results show that the communication in the WSN is the bottleneck, rather than the cryptographic processing at the devices, which can be observed in larger networks. This behavior shows that the overall performance is not significantly impacted by the cryptographic operations executed at the IoT device (sensors).

The remaining of this paper is organized as follows. Section 2 discusses the main related work. Section 3 describes the PROTeCt architecture in detail. Section 4 presents a discussion about PROTeCt. An analytical analysis about the considered architectures and solutions is performed in Section 5, while a experimental evaluation is presented in Section 6. Finally, Section 7 concludes this work.

## 2. Related Work

As the IoT paradigm gains maturity and its usage increases, the amount of data generated and the number of devices to manage becomes a challenge. Moreover, in order for it to be part of our everyday life, it is necessary that it is easy to use. The main purpose of the cloud computing and IoT integration is to provide the cloud’s storage, processing, scalability and networking capabilities to the severely-constrained IoT environments.

The integration of IoT networks with the cloud has been studied in some works, with most contributions focusing on providing tools to collect and store data generated by IoT devices, controlling access to this data and managing IoT devices. OpenIoT [11] is an open source middleware designed to facilitate the development and management of IoT networks. It provides a means to manage collected data from different types of IoT devices by utilizing an virtual sensor abstraction. The data collected is streamed to the cloud, where it can be explored with the assistance of semantic annotations. OpenIoT also provides a set of cloud services that offer several functionalities for the user, such as data visualization and IoT platform monitoring. OpenIoT does not define communication protocols among its components. On the IoT sphere, it wraps the communication, enabling the use of several protocols. Although secure communication can be achieved in all spheres of the architecture, user privacy is not a major factor, since data stored is accessible by the cloud platform.

The IoTCloud [4] is an open source platform that provides management of the IoT devices in the cloud. Its architecture is similar to OpenIoT, where IoT devices communicate through a message broker which is managed by a controller that is responsible for coordinating the entire platform. Applications query sensor data through a Web Services API exposed by the controller. Although security is not a major concern, IoTCloud utilizes several technologies through its architecture, and all of them have secure communications capability. IoTCloud does not offer cloud storage, and applications query live sensor data, which means that the user must pay attention only to how applications handle his data, not the cloud platform (IoTCloud).

Xively [12] is a proprietary cloud platform that manages IoT devices’ data. Users associate IoT devices with the platform and then can use several available services to consume the data or even build a specific service through their available APIs. IoT devices securely communicate with Xively servers through the MQTT [13] and TLS [14] protocols Libraries in several programming languages are available for MQTT, but only in C and Python for TLS. IoT data is stored in a time series database hosted on Xively’s cloud; however, users can replace Xively’s databases and send data to external databases, which can improve privacy levels, since user data can be stored in a trusted database. Xively’s platform provides advanced tools to manage IoT devices and its data, but the security algorithms used are not designed for severely-constrained IoT devices (such as wireless sensor networks), decreasing the coverage of its services. Concerning privacy, although users can store their data in external databases, this approach has two drawbacks: users must have the expertise and assets necessary to deploy a database, and even though data is stored externally, it still goes through Xively’s servers, which means that it has access to it.

The abovementioned works offer cloud platforms to manage IoT devices and their generated data, providing a means to use the collected data on already available applications and APIs. This approach is gaining traction since it has several benefits, such as the ease of having all IoT devices managed from one place and a way to consume the data with pre-available services. However, as already mentioned, the storage of user sensitive data in the cloud creates a privacy issue, where user data is exposed to several entities (cloud platform and services). SensorCloud [6] tackles this issue by implementing a cloud-based platform where only services that consume the data have access to it. A layer-based architecture is implemented, where IoT networks (or Wireless Sensor Networks) connect to the cloud through trust points (gateways), which are responsible for securely communicating with the cloud. Trust points encrypt sensor data with a secret key, and the data is stored in an encrypted form in the cloud platform. Services that will consume this data receive the private key, thus only authorized entities have access to the data, increasing users’ privacy level. The communication between IoT devices and trust points is not addressed by this architecture—only communication among the Trust Point and Cloud Provider/Services. SensorCloud envisions an architecture where users have IoT networks communicating to a trust point (TP), which is more resourceful and is also in each user’s possession. In order to use cloud services, the user binds his TP to a cloud provider, which stores the IoT data and provides a market place where the user can choose which services will consume his data. When sending data to the cloud, the TP encrypts it with a symmetric key, which is encrypted with the public keys of services with authorization to access the data, finally, the encrypted keys are stored in a key depot in the cloud provider. This approach prevents the cloud provider from accessing user data, providing a robust level of privacy, but requires the use of gateway on the users side, hindering adoption. Since it imposes the acquisition of another device (that must be more robust).

User-driven Privacy Enforcement for Cloud-based Services in the Internet of Things (UPECSI) [8] extends SensorCloud by implementing a privacy wide user-developer integrated solution. A privacy development language (PDL) was conceived to facilitate the development of cloud services. Developers use the PDL to provide detailed information about how the data should be handled by the application. This feature is then used by the users to allow or deny features of the application based on their privacy wishes. The security between the IoT networks and the cloud is achieved by a multi-layered architecture where privacy enforcement points (PEP), which are gateways at the border of the IoT, are responsible for security and privacy enforcement.

The implementation of security mechanisms in IoT devices presents several challenges, such as the location disclosure of mobile IoT devices [15] and others mainly related to the constrained capabilities of devices that compose IoT networks. Kothmayr et al. [16] presented an evaluation of the Datagram Transport Layer Security (DTLS) [17] protocol, which provides a secure channel utilizing UDP. The use of DTLS in constrained platforms is being actively studied. Results show that, although promising, this protocol needs some adjustments in order to fit the constrained characteristics of many IoT devices. Zhang et. al. [18] evaluated several implementations of the Advanced Encryption System (AES) cryptography system, which is the standard protocol utilized in the Internet. A severely-constrained platform was utilized, with a micro-controller of only 8 MHz, 128 KB of RAM memory and 512 KB of ROM memory. Delay and energy consumption were evaluated, and the results showed that, although the impact is not negligible, constrained platforms could utilize cryptography schemes.

## 3. PROTeCt: Privacy Architecture for Integration of Internet of Things and Cloud Computing

The UPECSI [8] architecture covers a wide range of aspects regarding the integration of IoT and cloud computing, from the development of a cloud service to the communication protocols between the border of the IoT network and the cloud platform. However, there is no mention of the internal communication of the IoT network, leaving this area unprotected. The PROTeCt architecture aims to fill this gap by introducing the necessary features to implement a secure communication scheme from the IoT devices to the cloud platform, the remaining part of the architecture is inherited from UPECSI.

In comparison with UPECSI, PROTeCt presents the following advantages: (1) it improves the fault tolerance of the system since it removes the gateway, which is a single point of failure; (2) it improves the security of the system, since it removes a component that is responsible for all security tasks and consequently, could impair the security properties of the system once compromised by a successful attack; and (3) by executing the security and privacy enforcement at the IoT devices, it is possible to implement fine grained control over the data, since each device could adopt a different security policy. These benefits come with a penalty related to the overhead added to the constrained devices. In fact, one of the goals of this work is to measure this overload. As already mentioned, gateways continue to exist in the network, but in PROTeCt, they do not execute any security task.

**Figure 1:**
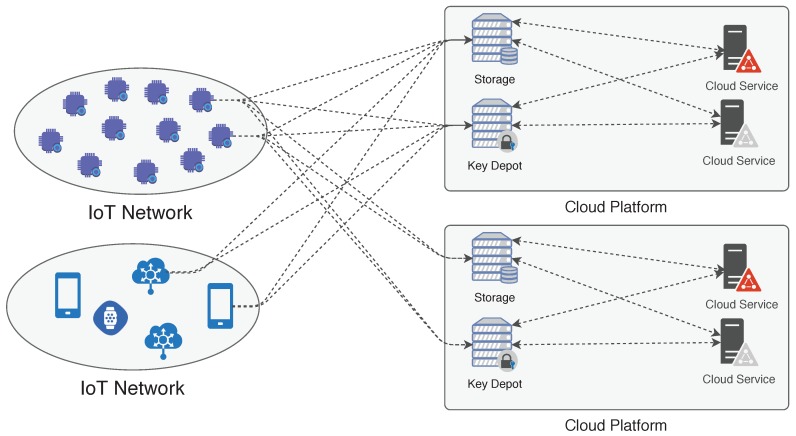
PROTeCt (**P**rivacy a**R**quitecture for integrati**O**n of internet of **T**hings and **C**loud computing): Internet of Things (IoT) devices send encrypted data to one or more cloud platform, and authorized cloud services access it to provide useful applications to users.

Figure 1 presents an overview of the PROTeCt architecture, where an user may have several IoT networks at his control. In a nutshell, first, the user must bind their IoT devices to the cloud provider (Section 3.1), enabling data transfer. Devices securely send data to the cloud storage Section 3.2. The authorized cloud services can process/access the user data according to the privacy policies Section 3.3. The architecture also contains a trusted third party that is responsible for providing default privacy policies and assisting in some security tasks in order to decrease device overload, as described in Section 3.3.2. The IoT network can also upload data to several different cloud platforms in order to increase the architecture fault tolerance or create a backup.

According to the privacy policy, the data generated by IoT devices (1) are not sent to the cloud; (2) are encrypted and sent to the cloud; or (3) are sent without any encryption processing. Afterwards, privacy requirements are held by the cloud services, which have the keys necessary to access only the data for which they are authorized. Some overhead is introduced by PROTeCt in the devices only when encrypting the data before transmission; thus, the analyzed scenarios will always consider that the privacy policy indicates the transmission of encrypted data. A detailed description of the PROTeCt is presented in the next subsections.

### 3.1. Binding of IoT Devices to the Cloud Platform

Users must first register an account in order to use the cloud service. After this, users can then bind their IoT devices to store the generated data. The binding process is achieved by using the OAuth 2.0 protocol [19], which is an open source and secure authorization protocol. After the binding process, the IoT devices can upload their data without possession of the user’s credentials.

The OAuth 2.0 protocol requires secure communication in order to provide really secure authorization. On the Internet, this is achieved by the use of the TLS protocol, which is used together with TCP. Since TCP cannot be used in IoT networks, another solution must be employed. This area is currently under active research, and there are already many proposals for security in the IoT transport layer. Particularly interesting is an adaptation of the Datagram Transport Layer Security (DTLS) [17] that is being studied by the DTLS In Constrained Environments (DICE) working group [20]. PROTeCt does not define a standard protocol for secure communication. Although a better suited secure channel protocol would present better performance, since this is an one time procedure, the use of DTLS does not impose a significant impact in the IoT network’s lifetime or performance.

The user can bind one device at a time or multiple devices at once. The process of binding multiple devices at once is only achieved when using a gateway. In this way, all IoT devices will share the same identification, and thus, the user will not be able to uniquely configure each device. OAuth’s Authorization Code Grant permission is used; this permission type requires user intervention only at the setup phase. After authorization, devices can upload data to the cloud without carrying the user credentials—only an Access Token is necessary, which is granted by device by the OAuth protocol.

**Figure 2:**
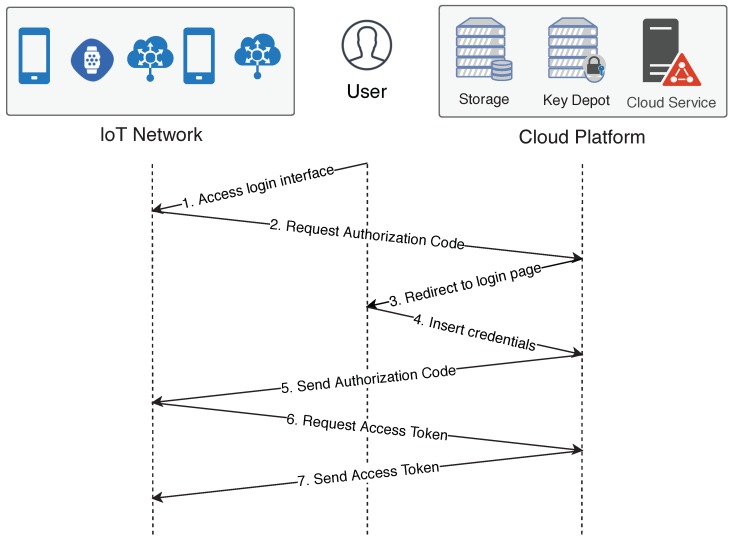
Binding scheme: The arrows represent the direction of communication among the IoT network, user and cloud platform. The numbers represent the order of communication and the corresponding steps.

Figure 2 presents the binding process for one device at time which is executed as following:

1. The user starts the process as the resource owner by entering device’s binding web page, where he must enter its credentials;
2. The device requests an authorization code for the cloud platform;
3. The cloud platform redirects the user to its login interface;
4. The user inserts its credentials;
5. After successfully receiving the user’s credentials, the cloud platform sends the authorization code to the device;
6. In possession of the authorization code, the device requests an access token to the cloud;
7. The cloud platform delivers the access token.

At the end of the binding process the device has an access token that must be attached in every data sent to the cloud, and the cloud platform has the device’s identification. The process is the same when binding multiple devices at once, the only difference is that at the end the gateway sends the received access token to all devices. Consequently, by using this method, the user cannot identify which device generated a specific data.

### 3.2. Privacy Policy Enforcement

A privacy policy (PP) defines how data can be stored in the cloud (encrypted, plain, or even not stored at all), which services can access this data and what the authorized services can do with it. Different from common privacy policies, where the user can only accept or deny all of it, in PROTeCt, the user can change it over time, accepting just parts of it. In this way, the user can assume real control over which data it sends to the cloud, knowing exactly what the service will do with it. Through the Privacy Development Language (PDL), cloud services provide an interface for users to see what data is used and for what purpose; the users can enable or disable specific features in order to restrict the access to their data. As example, users can enable or disable their location monitoring by a given cloud service and still use other features of the same service.

The PP is enforced every time an IoT device sends data to the cloud. The enforcement is done by the IoT device itself. When the user authorizes a new cloud service, a privacy policy associated with it is sent to all user devices. The same process occurs when updating a PP:

1. The user updates the PP through the cloud service interface;
2. The cloud service sends the updated PP to the trusted third party (TTP), which audits the information and generates a privacy configuration (CP), that specifies what devices will do to the data;
3. The TTP sends the new CP to all devices that provide data to the cloud service and need to update their policies.

### 3.3. Private Storage

After binding devices to a cloud platform and setting specific privacy policies for the authorized services, it is necessary to ensure that IoT data is stored and accessed only by authorized entities. These goals are achieved by a cryptography-based private storage scheme. As in the binding process, the communication between IoT devices and cloud must be secure, which is achieved by a proper transport layer protocol (this subject is further discussed in Section 4).

Before sending data to the cloud, an IoT device must filter it according to the privacy configuration, deciding if data must leave the user control sphere. The data is then encrypted with a symmetric algorithm. The key used by the symmetric algorithm is encrypted with the public key of the authorized cloud service and is stored in a key depot that is also in the cloud platform (as designed by [21]). In case there is more than one cloud service authorized to access the same data, the symmetric key must be encrypted once for each service. The data is stored only once in the cloud platform.

The symmetric key is updated regularly, preventing new services from accessing data stored previously to its authorization or revoked services to continue to access data. Past keys remain in the key depot, enabling access to data previously stored when desired. Cryptography operations can significantly decrease the IoT network lifetime. Since IoT is composed by the most diverse types of devices, it is important that the architecture encompasses as many types as possible. To try and reduce the cryptographic operations executed at the IoT devices, PROTeCt proposes two different approaches for key management: in the first one, the IoT devices manage cryptography keys (Section 3.3.1) and in the second one, these keys are managed by a trusted third party (Section 3.3.2). In the following text, these approaches are presented, and a discussion about the costs and benefits of each of them is further presented in Section 4.

#### 3.3.1. Keys Management by IoT Devices

**Figure 3:**
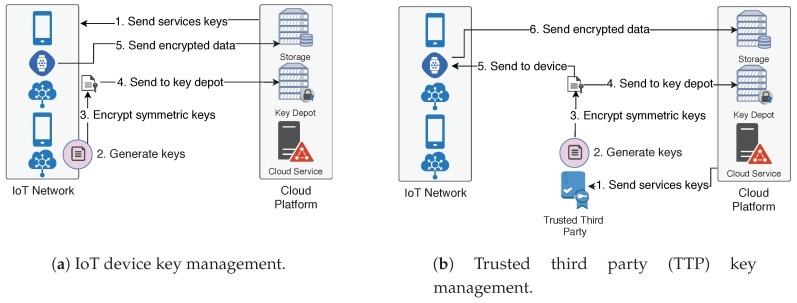
Key management scheme steps. The arrows represent the direction of interaction among the entities. The numbers represent the order and the corresponding step.

Figure 3a presents the approach where the device itself is responsible for generating symmetric keys, encrypting them with the cloud service public keys and sending them to the key depot in the cloud platform. This approach is intended for IoT devices that have enough computational and power capability to execute the following tasks without significantly decreasing their lifetimes:

1. Receive services’ public keys from the cloud platform;
2. Periodically generate symmetric keys;
3. Encrypt each new symmetric key once for each authorized cloud service;
4. Send the encrypted symmetric key to the key depot;
5. Send encrypted data to the cloud platform.

#### 3.3.2. Keys Management by a Trusted Third Party

Figure 3b presents the second approach, where a trusted third party (TTP) is responsible for some tasks related to key management, relieving the constrained devices from some tasks. This approach is useful for devices with constrained capabilities, where the tasks shifted to the TTP would significantly increase their lifetimes. This approach has the following steps:

1. **Cloud Platform**: Send services’ public keys to TTP;
2. **TTP**: Periodically generate symmetric keys;
3. **TTP**: Encrypt each new symmetric key once for each authorized cloud service;
4. **TTP**: Send the encrypted symmetric key to the key depot;
5. **TTP/IoT Device**: Securely send the symmetric keys to the IoT devices (using a transport layer security protocol as discussed at Section 3.1);
6. **IoT Device**: Send encrypted data to the cloud platform.

### 3.4. Flexible Privacy Policies

The data control scheme provided by the proposed architecture enables only previously authorized entities to handle the user data in the cloud. However, in some exceptional situations, it is convenient to decrease privacy requirements in order to allow new entities access to data. As an example, when a health monitoring IoT network detects an emergency, it could be more beneficial to the user if any medical personal could have access to his data, increasing the chances of getting assistance.

In order to attend the scenario above described, this work proposes the use of flexible privacy policies (FPP), which are secondary PPs that are only enabled when a given event is detected. The user must previously set the thresholds that will trigger the FPP, which can be based on IoT network generated data or even in external events. FPPs are created following the same scheme as common PPs (Section 3.2).

**Figure 4:**
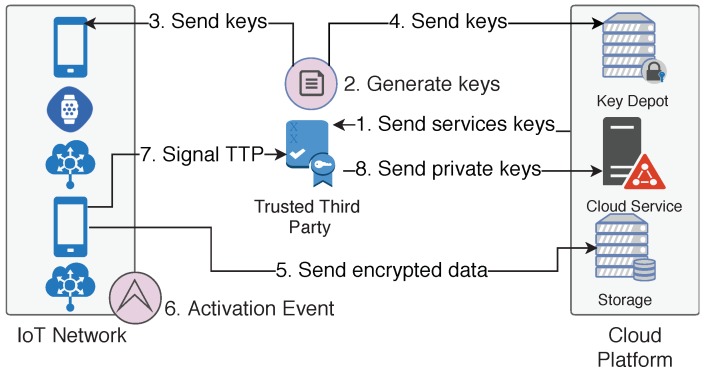
Flexible privacy policy activation scheme. The arrows represent the direction of interaction among the entities. The numbers represent the order and the corresponding steps.

Figure 4 shows how FPPs are activated. The scheme is similar to regular access control for common data, but in this case, the public keys used to encrypt symmetric keys are generated by the TTP, not by the services, and cloud services can access user data only after FPP activation. The FPP processing has the following steps:

1. The cloud platform sends services public keys to the TTP;
2. TTP generates cryptographic keys as follows:
  (a) Symmetric keys, used by IoT devices to encrypt their generated data;
  (b) Public/private key pair for each cloud service that will be granted access used by the TTP itself to manage the data access;
3. TTP securely sends (using a transport layer security protocol as discussed at Section 3.1) the symmetric keys to IoT devices;
4. TTP sends the symmetric keys encrypted with the public key (generated at step 2.b) to the key depot;
5. IoT device sends encrypted data to the cloud;
6. IoT device detects an event that activates the FPP;
7. IoT device signals the TTP;
8. TTP sends, through the key depot, the private key to the cloud service that must gain access to data. This private key is encrypted with the service public key (received at step 1).

After a flexible privacy policy is deactivated, the process must be re-executed (steps 2 to 5), so that the services activated by the FPP cease access to the data after it ends.

### 3.5. Enhanced PROTeCt

Enhanced PROTeCt (E-PROTeCt) improves the performance of PROTeCt by improving the costs associated with cryptographic processing and data transmission from the IoT devices to the cloud platform. These tasks occur frequently and therefore they impact the overall system performance. Other tasks, such as key update/management, do not occur frequently. As previously described, in PROTeCt the use of a secure transport layer protocol to send data to the cloud platform is proposed, and since the data is already encrypted at the application layer (by the private storage scheme), it is encrypted once more in the transport layer.

The data transmission protocol requires a secure channel between IoT devices and the cloud platform in order to constraint malicious actions (e.g., it prevents malicious entities from impersonating a user device by using its access token and sending fake data to the cloud provider). E-PROTeCt proposes an application layer secure communication scheme between IoT devices and the cloud platform. E-PROTeCt assumes there is a shared secret between each IoT device and the cloud platform which can be achieved in the binding process that also requires a secure communication (Section 3.1. Notice that since the binding process does not occur frequently, there is no meaningful performance impact of using a secure transport layer protocol for this stage.

**Figure 5:**
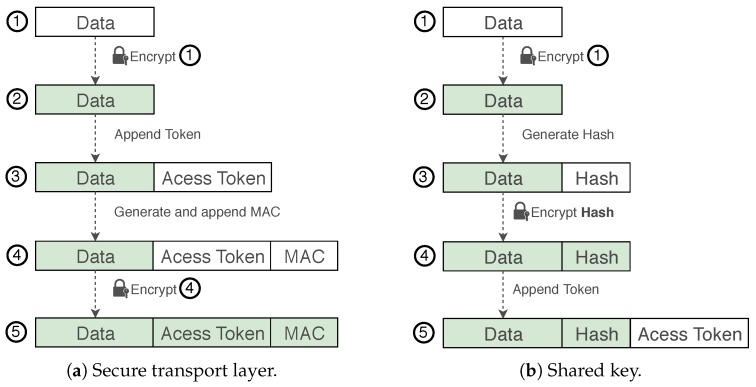
Cryptographic operations performed when utilizing a secure transport layer protocol (**a**) or a shared key with the cloud provider (**b**). The grey and white boxes represent encrypted and plain content, respectively.

Every transmission from an IoT device to the cloud platform must contain the access token (that identifies the device and proves it has permission to store data on behalf of an user) and the data itself (that has already been encrypted to be used only by authorized services). Figure 5a and Equation (1) show the cryptographic operations and message format of transmission when using a secure transport layer protocol. In this case, the DTLS protocol is used for reference (the MAC field stands for the message authentication code utilized by this standard) [17]. Data generated by IoT devices are encrypted twice—firstly, at the application layer by the PROTeCt private storage scheme (*Encrypt* ➀) which encrypts the data generated by the device; the secure transport layer protocol is responsible for the second encryption (*Encrypt* ➃) where the whole message is encrypted, including the already encrypted data, the MAC and the access token.

(1) Packet(x)=Encryption(Encryption(x)+AccessToken+MAC)

E-PROTeCt decreases the amount of data that must be encrypted in order to secure the data transmitted by IoT devices. Consequently, the amount of data that must be transmitted also decreases. The encryption performed at the application layer is always necessary, since this is how data must be stored in the cloud—in a way that only authorized cloud services candecrypt it. Therefore, it is necessary to design a method to provide data authenticity at the application level, removing the secure transport layer protocol.

Since the data is already encrypted for private storage, it is necessary to define a way to associate the access token with the encrypted data, preventing impersonation by simply observing a transmission and sending other data with the observed access token. E-PROTeCt proposes the transmission of a hash of the encrypted data, which should be encrypted with the shared secret (symmetric key) between the IoT device and the cloud provider. In this approach, even by knowing the access token, an attacker is not able to fabricate a message, since it does not have the shared secret to encrypt the hash.

Figure 5b and Equation (2) show the E-PROTeCt cryptographic operations and message format, where data to be stored in the cloud platform is encrypted only once (*Encrypt* ➀), and then the shared symmetric key is utilized to encrypt the hash of the encrypted data (*Encrypt Hash*). Afterwards, the access token is attached, and the message is transmitted. The cloud platform verifies the authenticity of received data by (1) identifying the matching symmetric key for the access token; (2) decrypting the hash portion of the message; and finally, (3) comparing it with its own hash computed over the received encrypted data. It is important to note that the hash is obtained from the encrypted data, and the cloud platform does not have access to the corresponding plaintext:

(2) Packet(x)=α+Encryption(Hash(α))+AccessToken,whereα=Encryption(x).

## 4. Discussion

This section presents a detailed discussion of the proposed architecture. First, we discuss the aspects related to secure wireless communications for constrained devices, and how it compares to the technologies utilized in full fledge devices, such as the privacy enforcement point from UPECSI. Afterwards, we present an analysis regarding privacy enforcement and a discussion about the aspects of PROTeCt and E-PROTeCt, presenting their limitations and benefits.

### 4.1. Direct Communication between IoT Devices and the Internet

The proposed architecture aims to use communication protocols that are suited to the IoT devices’ specific characteristics and can communicate with standard Internet protocols. This approach enables the direct communication of IoT devices with common Internet devices, so that each IoT device is addressable and reachable from the Internet. The UPECSI architecture does not define the communication protocols between IoT devices and gateways. PROTeCt defines those protocols and transfers gateway responsibilities to IoT devices. Although gateways are not used to represent the entire IoT network, they can still be used for other functions, such as data forwarding and protocol translations.

Although our work defines communication protocols at the application layer, a discussion regarding the secure transport layer protocols designed for constrained devices is pertinent. Security at lower layers (network and link) for constrained IoT devices, although still presenting several challenges, is at a mature state with consolidated protocols: IEEE 802.15.4 for the Link layer, 6LoWPAN for IPv6 addressing and RPL [22] for routing.

Implementing security schemes in IoT constrained devices has several challenges. The additional load necessary for cryptographic operations can increase execution latency, decrease device lifetime (in cases where there is a limited energy source) and include some overhead on message exchange, both in quantity and size. Internet security standards, such as TLS, utilize the public key infrastructure (PKI) [23] to exchange symmetric keys, and then the communication is encrypted with a symmetric algorithm, similar to the approach used by this work. The first step, regarding key exchange, is especially expensive, both in terms of message exchange and CPU workload. Many works related to security for constrained devices assume the keys have been previously loaded into each device.

TLS supports several algorithms for key exchange, message confidentiality and authentication. The most common algorithm utilized for key exchange is RSA [24], which may impose a restrictive load on severely constrained devices. Therefore current studies advise the utilization of elliptic curve cryptography algorithms, such as Elliptic Curve Diffie–Hellman (ECDH) [25], which consumes less resources than RSA for 8-bit CPU [26]. In order to increase cryptographic operations performance and decrease energy consumption, it is common for IoT platforms to include a dedicated hardware to execute this task [27]. Although there have been several proposals to enable secure communications for constrained devices [20,28,29], the Internet Engineering Task Force (IETF) is still working on a standard.

Since the intent of the proposed architecture is to enable direct communication of IoT constrained devices with the Internet, which means using standard-based communication protocols, this work does not implement a new transport layer protocol or chooses one from the several proposals available. PROTeCt assumes a secure channel protocol, and the analysis conducted by this work utilizes DTLS as an implementation, while E-PROTeCt implements secure communication in the application layer, decreasing the penalties related to those mechanisms.

### 4.2. PROTeCt and E-PROTeCt Mechanisms

This section discusses the schemes presented in this work. First, a discussion about the binding protocol is presented, indicating its benefits and the possible difficulties of implementation into IoT devices. Afterwards, we discuss aspects related to how privacy policy is enforced by IoT devices, regarding their constrained characteristics. The private storage scheme is also debated. Thia is the protocol that is executed every time a device sends data to the cloud, and thus, its overhead must be analyzed in detail. Finally, the flexible privacy policy scheme is discussed.

#### 4.2.1. Binding

The OAuth 2.0 requires a secure communication channel, since it does not provide reliability for message exchange. In order to provide device specific binding, the user must access an interface provided by each device (Step 1 in Figure 2). This kind of service should be optimized in order to prevent too much load on the constrained devices. Nonetheless, the binding processing occurs only once—when the user is registering the device in a cloud platform. Therefore, the load that the binding scheme adds to the network can be neglected, and it is recommended that an established secure transport layer protocol, such as DTLS, is used.

#### 4.2.2. Privacy Policy Enforcement

Privacy policy enforcement is performed as in the UPECSI architecture, but instead of being executed by the gateway, it is executed by each IoT device. Updating a privacy policy is achieved through its respective cloud service, usually by a web interface or smartphone application. This scheme only introduces the reception and enforcement of privacy policies by the devices. Reception is unlikely to occur very often and enforcement is implemented by simple conditional statements. Consequently, these steps do not significantly increase a device’s load, being perfectly suitable for constrained devices.

#### 4.2.3. Private Storage

User privacy requirements are guaranteed by using cryptography. Private data is stored encrypted in cloud platforms, and only authorized cloud services have the keys to decrypt it. Although IoT devices must encrypt data going to the cloud, some operations could be relieved by shifting them to the trusted third party. For example, the key management could be performed by the TTP, leaving the IoT devices only with the tasks of receiving keys and encrypting data. This scheme is recommended for IoT networks where devices have very constrained resources.

IoT devices can be responsible for managing symmetric keys, excluding the TTP from the process. This method is more expensive to devices, which must execute tasks with high computational costs: symmetric key creation, asymmetric encryption of symmetric keys and sending encrypted keys to services. The encryption of symmetric keys is periodic and performed once for each service authorized to access the respective data, which means that these operations can be quite expensive if there are many services. This method must be used with caution. If the number of services and the periodicity of symmetric key generation are high, this can potentially compromise the IoT network’s lifetime and responsiveness. The advantage of using this method is that the TTP will not have the keys necessary to access data, therefore decreasing the surface of attack for the network.

#### 4.2.4. Flexible Privacy Policy

Flexible privacy policies were proposed to increase the architecture’s adaptability. This scheme enables the user to define boundaries where some cloud services can begin or stop having access to private data. This is especially useful in exceptional situations where it is more beneficial to give up privacy then keep it.

Since the main purpose of FPPs is use in exceptional situations, this may include cases where the IoT device itself ceases functioning. In such cases, it is only possible to activate the FPP by an external agent. To ensure the proper behavior in this situation, the TTP is responsible for enforcing the FPP, managing keys and granting access to services when necessary. This method adds more reliability to the scheme. The load introduced by this feature is only related to triggering the FPP by signaling the TTP when a FPP must be activated (by an external agent or an IoT device), thus requiring very little resources for a very useful feature.

### 4.3. Privacy Analysis

User privacy requirements are guaranteed by using cryptography in all stages of the data life cycle. Through the PDL, the cloud services are required to inform the user about the operations performed in the consumed data. This approach allows the user to make conscious choices about what services and features to use regarding its privacy requirements.

Access to users’ information is limited to only the necessary entities by storing it in an encrypted form. In this approach, not even the cloud platform has access to data. In order to implement encrypted storage, a key management scheme is applied. PROTeCt and E-PROTeCt improve this scheme by transferring the responsibility of key management and data encryption from the gateway to the IoT devices. This approach removes a single point of failure and decreases the attack surface.

Although the main goal is the same for UPECSI, PROTeCt and E-PROTeCt, Table 1 shows significant differences among these architectures. UPECSI does not define communication protocols between the IoT devices and the gateway, while PROTeCt and E-PROTeCt do, since the privacy enforcement is performed at them. The overhead introduced by the PROTeCt and E-PROTeCt approaches can be split into two segments: privacy enforcement, mainly related to cryptographic functions executed by IoT devices (Section 4.2), and secure communication (Section 4.1). The latter is not only related to the architecture goal but to any approach that implements secure communication. In this case, although UPECSI does not define communication at the IoT layer, any implementation must secure this layer as well.

## 5. Analytical Analysis

In order to provide secure communication inside the IoT network, PROTeCt and E-PROTeCt increase the load on IoT devices, (1) due to the insertion of cryptographic operations inside these devices and (2) by increasing the amount of transmitted data. The following sections present an analytical analysis of these two aspects. Four approaches are evaluated: (I) *No Security* in which data is transmitted from IoT devices to the cloud platform without any secure protection (this approach is used as the baseline); (II) *UPECSI* [8] in which data is transmitted from the IoT device to the gateway without any secure mechanism, and the gateway protects data using standard transport layer security (TLS) and sends it to the cloud; (III) *PROTeCt* where IoT devices encrypt data and send it to the cloud platform through a secure transport layer (DTLS); and (IV)*E-PROTeCt* where IoT devices secure the communication by encrypting a hash of the encrypted data with a symmetric key that is securely shared with the cloud platform (Section 3.5).

### 5.1. Cryptographic Operations Executed by IoT Devices and Gateway

Table 2 presents an analysis of the cryptographic operations performed in each approach with consideration of the amount of bytes encrypted by both the IoT devices and the gateway. The following nomenclature is used: C(u) is the cost of encrypting *u* bytes; T(j) is the resulting size of the encryption of *j* bytes; *x* is the size of data generated by IoT devices that must be protected; *y* is the site of the access token used to identify the IoT device; and *z* is the size of the hash.

Obviously, the solution without security has no encryptions in both places. The UPECSI approach does not define any security mechanism between a IoT device and gateway; thus, there are no cryptographic operations inside the devices. All security is enforced by the gateway that first encrypts the IoT data (C(x)) and then, encrypts the whole application message plus a MAC (we consider the MAC size to be equal to the hash size, since it represents a hash over the message that should be authenticated) by using a secure transport layer protocol (C(T(x)+y+z+4)). The 4 bytes are related to an overhead that is necessary to represent application data, for example, using the Constrained Application Protocol (COAP) protocol [30].

The PROTeCt approach implements a secure channel in the IoT device, thus moving all cryptographic operations from the gateway to the IoT devices. In this case, the gateway is only responsible for forwarding messages and does not perform a single encryption. Finally, E-PROTeCt reduces the amount of cryptography by securing the communication at the application layer. After encrypting the generated data (C(x)), it needs to encrypt only the hash over the result, drastically reducing the amount of encryptions performed by these constrained devices.

The three approaches with security require two cryptographic operations but for different amounts of data. In UPECSI and PROTeCt, both encryptions depend on the amount of data generated by IoT devices (*x*), while in E-PROTeCt, only one of the encryptions depends on this value, since C(z) is always the same size. Moreover, E-PROTeCt does not encrypt data twice, reducing the amount of data that must be encrypted.

**Figure 6:**
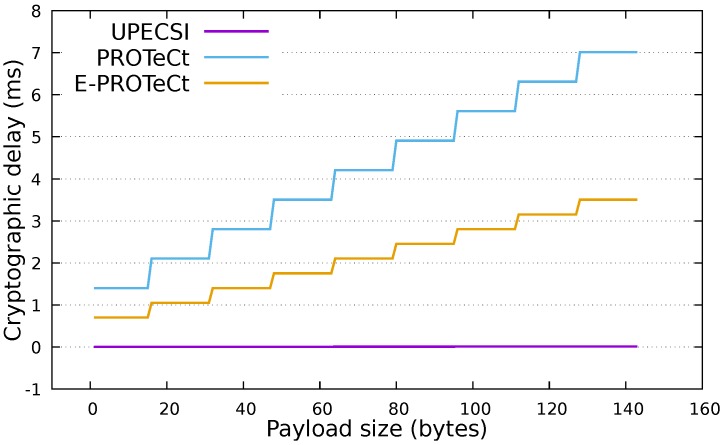
Cryptographic delay for the analyzed architectures.

Figure 6 presents the delay in milliseconds introduced by the cryptographic operations for different packet sizes. This analysis was performed to consider the time necessary for encrypting the data with the AES-CBC encryption algorithm using a key of 128 bits. The delay was calculated with consideration of the encryption time in a Micaz Mote, a constrained device utilized for wireless sensor networks [18]. The steps observed in the graph are related to the padding used by the encryption algorithm, as discussed in the next section. The delay at the gateway (for the UPECSI architecture) was observed in a Raspberry Pi device [31] which presented a negligible value.

### 5.2. Amount of Transmitted Data

In networks with severely-constrained devices, a reduction of a few bytes can generate a significant increase in the lifetime of the network, since usually, the amount of transmitted data is very small. It is important to note that the cryptography scheme utilized has a direct impact on this item. In this work, we consider the AES scheme in CBC mode which requires that all encrypted data must be padded to match the block size, which is 128 bits (16 bytes). For example, the encryption of 10 bytes of data using AES-CBC will result in 16 bytes. This is specially relevant to IoT networks where the size of transmitted data is usually very small which makes the padding a quite relevant overhead. The size of the hash also deserves a remark—when using standard protocols (UPECSI and PROTeCt), the hash is actually a MAC, and its size is 20 bytes. In E-PROTeCt, the hash could be smaller, since it is only used to identify the encrypted message.

Table 3 shows that in PROTeCt and E-PROTeCt, IoT devices send significantly more data than in UPECSI and No Security. In fact, in these approaches, the security mechanisms are implemented by the IoT devices. Moreover, in these approaches the gateway only forwards the information received from the IoT devices, while in UPECSI, it applies the security mechanisms and sends the resulting information to the cloud provider.

**Figure 7:**
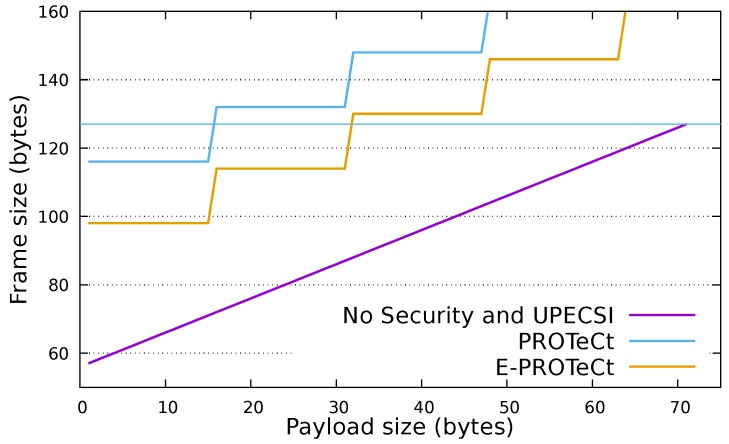
Overhead in the frame size for each approach.

Figure 7 presents the total packet size for varying sizes of payload for each of the evaluated approaches. For this analysis the following item sizes were used: 10 bytes for the hash, 10 bytes for the access token, 20 bytes for the MAC and 52 bytes for the network stack headers. The horizontal line shows the maximum frame size allowed by the IEEE 802.15.4 which is 127 bytes. Above this line the packet should be fragmented, increasing the communication overhead. As can be seen, the PROTeCt and E-PROTeCt approaches introduce a significant amount of overhead which limits the payload size that can be transmitted in a single frame. No Security and UPECSI present the same total packet size, since both approaches do not apply security in the IoT communication. When using the secure approaches, the maximum supported payload for a single packet is 15 bytes for PROTeCt and 31 bytes for E-PROTeCt; in the insecure approaches, it is 75 bytes.

## 6. Experimental Evaluation

This section presents the simulation scenario and the results obtained and evaluates the energy consumption (device life time) and end-to-end delay of communication between IoT devices and the cloud provider.

### 6.1. Experimental Setup

**Figure 8:**
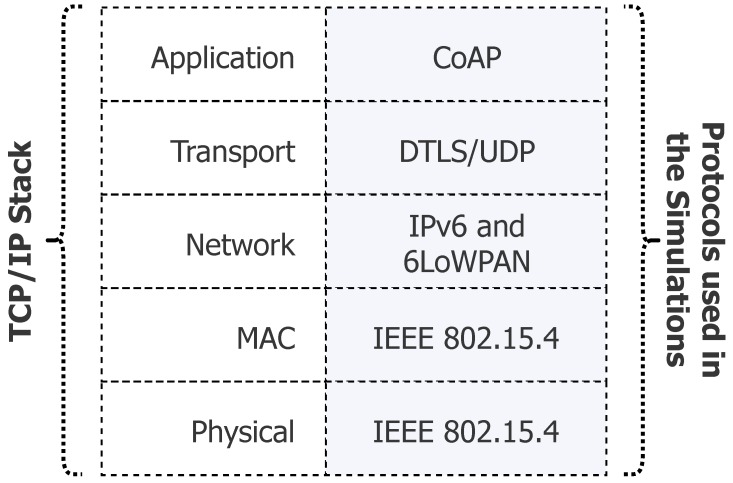
Network stack.

The evaluation was conducted through simulations and executed using the ns-3 simulator, considering the four previously-described approaches. In order to obtain a relevant amount of data, several scenarios were simulated by varying the number of IoT devices in the user networks and the frequency of data generated by those devices. Figure 8 presents the protocol stack used by the devices; in all layers, standards developed specially for constrained devices were utilized.

The physical and MAC layers used the IEEE 802.15.4 standard [32], which works at 2.4 GHz with a bandwidth of 250 kbps and a maximum data frame of 127 bytes. Network addressing was achieved by IPv6 [33], together with 6LoWPAN [34] to decrease the header size. The transport layer used the UDP protocol [35], and for PROTeCt, the DTLS [17] was used to provide a secure channel. The COAP standard [30] was used in the application layer to represent data, introducing a 4-byte overhead. Together, the lower layers added 52 bytes of overhead, leaving only 75 bytes for the transport and application layers. Note that those characteristics represent a WSN, i.e., a network composed of very constrained devices.

**Figure 9:**
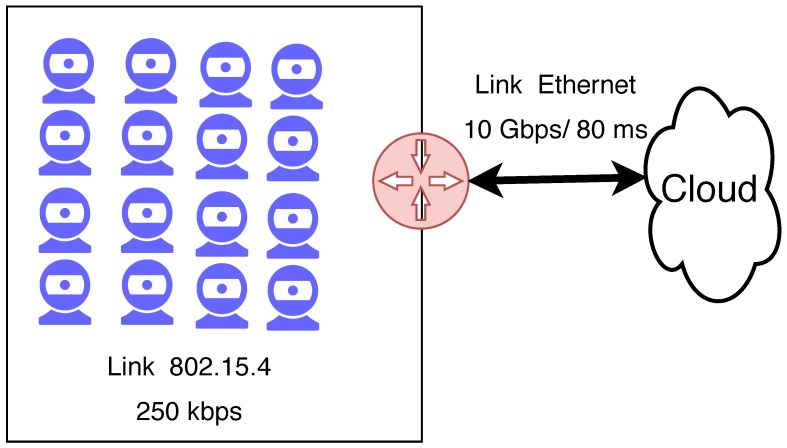
Scenario with 16 IoT devices.

All simulations were performed with 10 bytes of payload, which is enough to represent many values (e.g., the room temperature, the state of a lamp that could be on or off or the heart rate of a person). The frequency of data generation was varied between 15 s, 30 s, 1 min, 2 min, 3 min, 4 min and 5 min. Each packet was randomly generated within these periods. The number of IoT devices in the network was varied from 4 to 160 nodes. Devices were connected to a gateway which was connected to the cloud platform; this last link had a latency of 80 ms, simulating the unreliable nature of the Internet. Figure 9 presents the simulation scenario with 16 nodes. All IoT devices were in the communication range of the gateway, which means all communications inside the IoT network did not exceed one hop. There was no interference in the environment besides possible collisions among IoT devices.

In order to measure the energy consumption and delay when devices encrypt data, measurements from [18] were used, which can evaluate several AES implementations in a severely-constrained device. The Micaz Mote was used. It has an 8-bit ATmega128L microcontroller, 128 KB RAM, 512 KB ROM, a 2.4 GHz CC2420 [36] and a RF (radio frequency) transceiver that supports the IEEE 802.15.4 standard. Energy consumption for transmission and reception are referred to in its datasheet details [36]. The encryption delay for the gateway was obtained from a study conducted by Fisher et al. [31] that evaluated the AES protocol on the Raspberry Pi Model A [37] device. This device has an ARM processor of 700 MHz and 256 MB of RAM. Table 4 shows the delay and current drawn for cryptographic operations utilized during simulations. It also shows the current drawn by the radio of devices for the transmitting and receiving states. The values are related to the encryption of one block which corresponds to 16 bytes.

The following sub-sections presents an analysis of the delay and life time of IoT devices. Delay is regarded as the difference between the moment that a device generates data and the moment that the data is received by the cloud platform. The results shown are related to the average delay of all transmitted packets during the simulation; the standard deviation also is presented. The life time of a device is calculated as if it was powered by a 2600 mAh battery. The calculated energy consumption regards the transmission and reception of messages and the cryptographic operations performed by devices, and thus life time values are substantially higher than reality. The analysis only comprehends the most distinct aspects among the evaluated approaches. The cryptographic key is set up only once which means the keys do not change during the simulation.

### 6.2. Network Saturation Analysis

**Figure 10:**
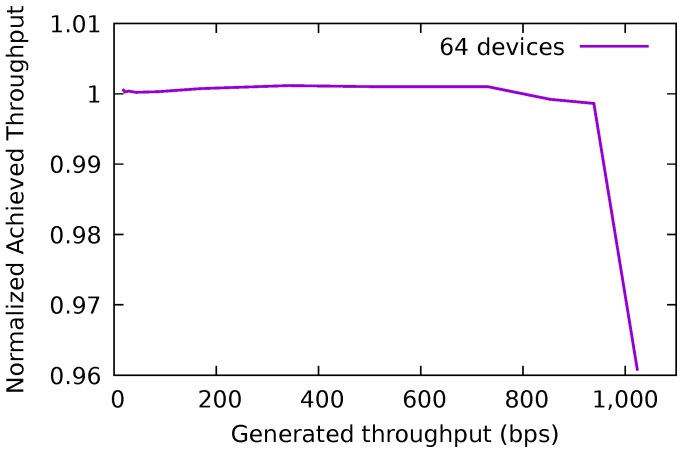
Ratio between the generated and achieved throughput—No Security approach with 64 devices.

An analysis was performed in order to identify the simulated scenarios that saturate the network, irrespective of the approaches being evaluated. Figure 10 presents the ratio between the generated and the achieved throughput for the No Security approach (which has no overhead) with 64 devices in the IoT network. When the throughput ratio drops below 1 it means that there is data that was generated but not received by the cloud platform, indicating the scenarios where the generated throughput was more than the network could handle. As can be seen, this situation occurred when the generated throughput was near 1000 bps. The following scenarios generate more data than the network can support: All simulations with 160 devices and the simulations with 64 and 128 devices that generated packets every 5 s.

### 6.3. Delay Analysis

**Figure 11:**
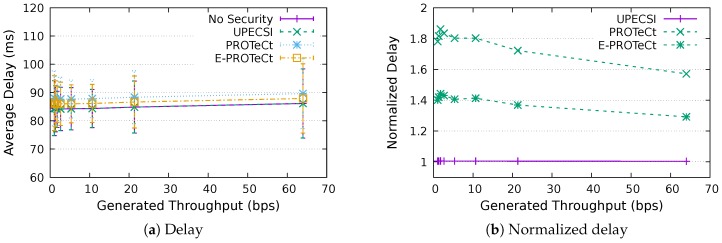
Delay analysis for an IoT network with four devices.

**Figure 12:**
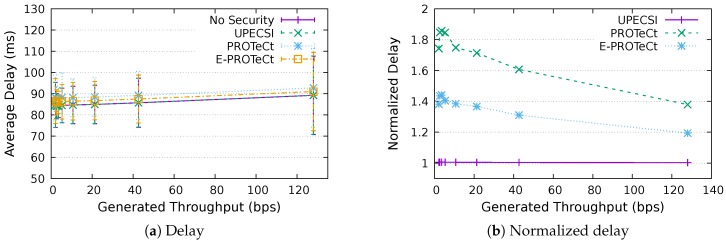
Delay analysis for an IoT network with eight devices.

**Figure 13:**
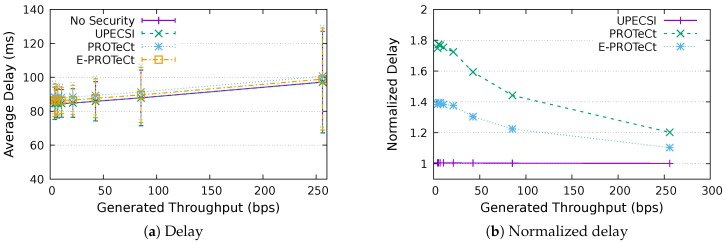
Delay analysis for an IoT network with 16 devices.

**Figure 14:**
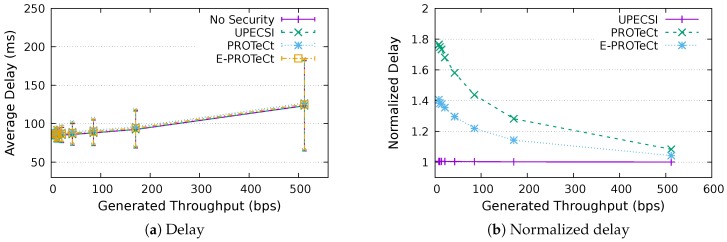
Delay analysis for an IoT network with 32 devices.

**Figure 15:**
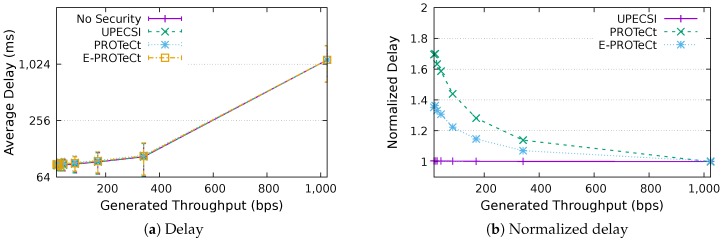
Delay analysis for an IoT network with 64 devices.

**Figure 16:**
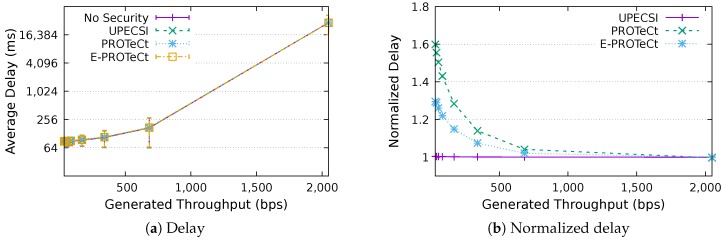
Delay analysis for an IoT network with 128 devices.

**Figure 17:**
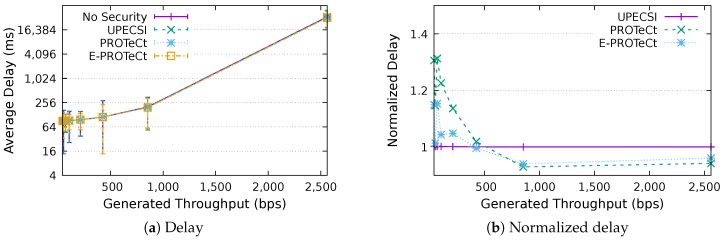
Delay analysis for an IoT network with 160 devices.

For the delay analysis of the four previously describe approaches, we varied the number of IoT devices in the network and the period of data generation, leading to different generated throughputs. The results for the delays according to the generated throughputs are presented in Figure 11, Figure 12, Figure 13, Figure 14, Figure 15, Figure 16 and Figure 17 for IoT networks with 4, 8, 16, 31, 64, 128 and 160 devices, respectively. The left graph of each figure presents the average delay, while the right graph presents the normalized values considering the *No Security* approach as the baseline. For the normalized graphs, the 80 ms delay from the gateway to the cloud link was not considered; thus, we could better evaluate the differences regarding only the IoT network.

The left graphs show that, in comparison with the gateway–cloud link delay of 80 ms, the IoT network delay had a much lower impact on the network’s performance. It is also possible to note that the network saturation point, presented in Section 6.2, holds for all approaches, indicating that the security features implemented in some of them do not hinder the network capacity.

In the graphs on the right-hand-side, it is possible to analyze the overhead introduced by each approach inside the IoT network. The UPECSI approach presents negligible overhead, since the encryptions are performed only at the gateway. The delay of PROTeCt and E-PROTeCt approaches is, at most, around 40% and 80% superior than the solution without security. The difference decreases as the number of devices in the IoT network increases. This happens because the overhead of cryptographic operations becomes less significant in comparison with the medium access algorithm delay; this fact is even more evident in Figure 17. In absolute terms, the delay differences between PROTeCt and E-PROTeCt and the solution without security were 3.5 ms and 1.75 ms, respectively. When the scenarios where the network is overloaded were not considered, the differences were almost constant, no matter the number of devices in the network or the amount of data generated by each of them. The delay overhead reduction when using E-PROTeCt (compared to PROTeCt) is 50%, indicating that the implementation of security mechanisms in the application layer offers considerable advantages in relation to the utilization of a secure transport layer protocol.

IoT networks with constrained devices are mainly used with a low data generation frequency, and in such casea, a delay overload of 1.75 ms can be regarded as viable, providing a great trade-off when considering the security of communications.

### 6.4. Life Time Analysis

**Figure 18:**
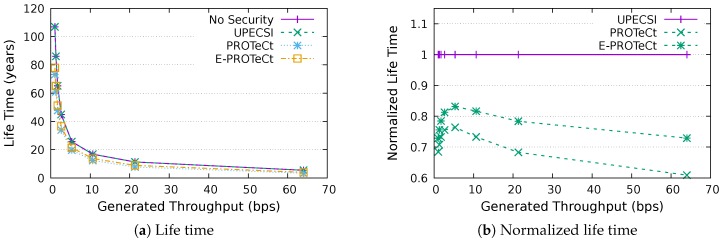
Life time analysis for an IoT network with four devices.

**Figure 19:**
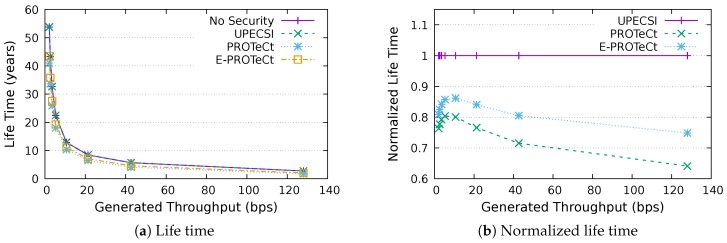
Life time analysis for an IoT network with eight devices.

**Figure 20:**
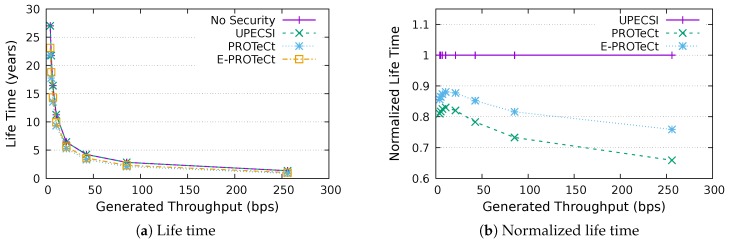
Life time analysis for an IoT network with 16 devices.

**Figure 21:**
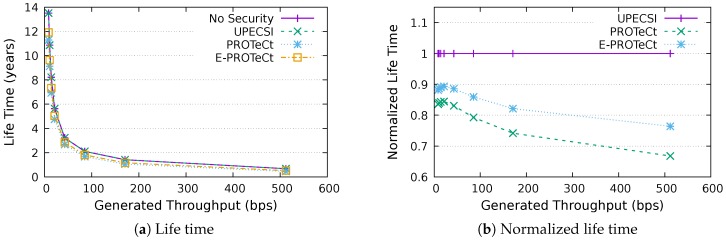
Life time analysis for an IoT network with 32 devices.

**Figure 22:**
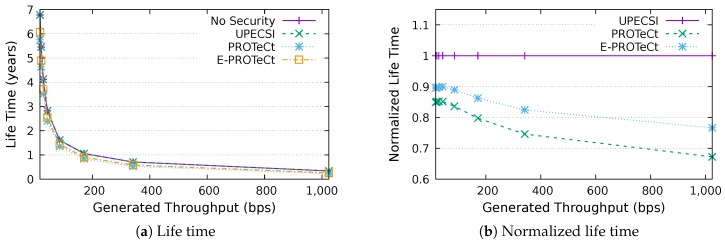
Life time analysis for an IoT network with 64 devices.

**Figure 23:**
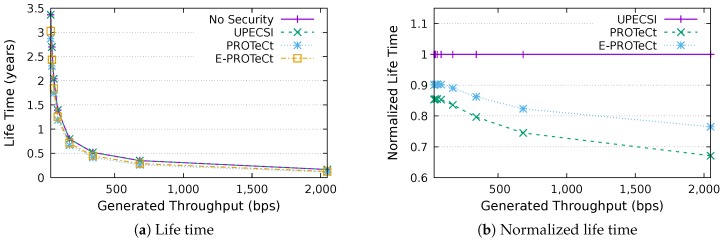
Life time analysis for an IoT network with 128 devices.

**Figure 24:**
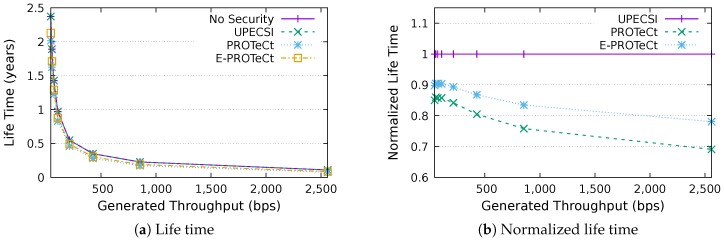
Life time analysis for an IoT network with 160 devices.

This section discusses the results for the device life time analysis, considering the same scenarios presented in the previous section. The results for the life time according to the generated throughputs are presented in Figure 18, Figure 19, Figure 20, Figure 21, Figure 22, Figure 23 and Figure 24 for IoT networks with 4, 8, 16, 31, 64, 128 and 160 devices, respectively. The left graph of each figure shows the life time in years according to the throughput generated by the IoT network, while the graphs on the right-hand-side present the normalized values considering the solution without security as a baseline.

The calculation of energy consumption is based on two values: the radio communication and the cryptographic operations. The first occurs in all approaches, while the second occurs only in PROTeCt and E-PROTeCt, which provide secure communication inside the IoT network. The radio’s energy consumption is always superior in comparison with the consumption necessary for cryptographic operations, and the difference between the secure and non-secure approaches increases linearly. Therefore, the impact on a devices life time caused by cryptographic operations processing is greater in simulations with low throughput.

As can be seen in the right graphs of each figure, a device’s life time decreases exponentially according to the generated throughput, which indicates that severely-constrained devices are suitable for applications with a low data rate. The decrease in a device’s life time is also proportional to the number of devices in the network, since the spectrum is shared, and all devices receive every packet (and then discard it when the destination MAC address does not match its own address). This factor can be mitigated (or even completely removed) if using a deterministic algorithm in the MAC layer, which depends on the network’s characteristics. Extremely high life time values were found, because several aspects that impact this metric were not measured. In this evaluation, we considered only the aspects that are different for each approach, i.e., radio communication and cryptographic operations, since the amount of transmitted data and cryptographic operations are different for each solution.

In the right-hand-side graphs of each figure, it is possible to identify the overhead introduced by each approach. As already commented, the solution without security was used as a baseline. The UPECSI has no overhead in comparison to the solution without security (gateway overhead does not impact this metric). On the other hand, PROTeCt and E-PROTeCt present some overhead in terms of life time. The former presented an average decrease of 21.80% in the network life time, while in the latter, the average decrease was 15.51%. The absolute life time ratio difference between PROTeCt and E-PROTeCt varied from 0.04% and 12.02%, leading to an average increase varying from 5.18% and 19.74% of E-PROTeCt over PROTeCt. Although less prominent than in the delay analysis, the results reported in this section suggest that E-PROTeCt can significantly reduce the impact caused by the execution of secure mechanisms in severely-constrained devices.

### 6.5. Lessons Learned

This section presents a few observations about the experimental evaluation.

1. In the saturation analysis, it was clear that the small throughput of the network, only 250 kbps, is a limiting factor that is reached long before a device’s processing limitations. Thus, the overhead at the devices originated from the PROTeCt and E-PROTeCt architectures is not the most significant limiting factor. All graphs show that the secure approaches never change the saturation threshold of the network, they only add some overhead.
2. Extremely-constrained devices, which are the focus of this work, have such a low bit rate that the gateway never becomes a bottleneck, which means that there is no drawback regarding performance in implementing security schemes at the gateway, such as UPECSI does. The drawback only relates to security, since this approach presents an unique point of failure.
3. Devices that does not have an unlimited power source must save energy at all costs. Even though the analyzed architecture does not change a network’s saturation threshold, they do marginally decrease its lifetime, which, in this case, could be a significant factor. It is also important to note the significance of a well suited medium access control protocol. In the simulations performed by this work, all devices received all messages transmitted into the medium. This approach caused an exponential decrease in a device’s lifetime related to the number of devices in the network. If a protocol that better suits constrained devices was utilized, the lifetime decrease could be far lower then that observed.

## 7. Conclusions

This work reported on our efforts to improve security and privacy in the integration of IoT and cloud computing. In the proposed solution, privacy is provided by a cryptography-based scheme where only the interested parties can access the data, i.e., data is stored in encrypted form, and only authorized users and cloud services have access to the keys necessary for the decryption. While UPECSI enforces privacy only from the IoT gateway, PROTeCt and E-PROTeCt enforce privacy from the IoT devices, improving the overall system security. E-PROTeCt tries to decrease PROTeCt’s overhead by implementing its own security schemes instead of using a secure protocol in the network layer. Both PROTeCt and E-PROTeCt were proposed with a focus on severely-constrained devices (mostly of them running on batteries) and therefore, must have a low overhead. The analysis conducted by this work evaluated all mentioned approaches in this kind of device both analytically and experimentally.

The analytical analysis evaluated the number of cryptographic operations and amount of data overhead necessary at IoT devices and gateway, while the experimental evaluation considered the delay and device lifetime. UPECSI presents no overhead at the devices, since data travels without any security inside the IoT network, while the overhead introduced by PROTeCt and E-PROTeCt shows that it is feasible to include security protection inside the IoT network, i.e., it is not necessary to trust the gateway. In general, the results obtained by this work show that enforcing security and privacy at the IoT device comes with a cost, but it is not prohibitive. In particular, for severely-constrained devices, the implementation of those features must be as optimized as possible.

## Figures and Tables

**Table 1:** Comparison among the architectures.

	UPECSI	PROTeCt	E-PROTeCt
**Privacy enforcement point**	Gateway	IoT device	IoT device
**Data protection**	Gateway-Cloud	IoT-Cloud	IoT-Cloud
**Secure communication**	Gateway-Cloud	IoT-Cloud	IoT-Cloud
**IoT device overhead**	None	Moderate	Low
**Single point of failure**	Gateway	None	None

**Table 2:** Cryptographic operations by IoT devices and the gateway, considering *x* bytes of data, an access token of *y* bytes and a hash of *z* bytes.

	Encryptions by IoT Devices	Encryptions by the Gateway
**No Security**	—	—
**UPECSI**	—	C(x)+C(T(x)+y+z+4)
**PROTeCt**	C(x)+C(T(x)+y+z+4)	—
**E-PROTeCt**	C(x)+C(z)	—

**Table 3:** Amount of data transmitted considering generated data of *x* bytes, an access token of *y* bytes and a hash of *z* bytes.

	IoT Device → Gateway	Gateway → Cloud Platform
**No Security**	*x*	x+y+4
**UPECSI**	*x*	T(T(x)+y+z+4)
**PROTeCt**	T(T(x)+y+z+4)	T(T(x)+y+z+4)
**E-PROTeCt**	T(x)+T(z)+y+4	T(x)+T(z)+y+4

**Table 4:** Delay (ms) and current drawn (mA) by constrained devices when encrypting 16 bytes of data using AES-128 [18] and current drawn for the RX and TX radio states.

Item	Value
**Encryption (delay)**	0.3506 ms
**Encryption (current drawn)**	25.501 mA
**Transmitting (TX)**	17.4 mA
**Receiving (RX)**	18.8 mA
